# Gene Expression Profiling Reveals Similarities between the Spatial Architectures of Postnatal Articular and Growth Plate Cartilage

**DOI:** 10.1371/journal.pone.0103061

**Published:** 2014-07-28

**Authors:** Michael Chau, Julian C. Lui, Ellie B. M. Landman, Stephan-Stanislaw Späth, Andrea Vortkamp, Jeffrey Baron, Ola Nilsson

**Affiliations:** 1 Pediatric Endocrinology Unit, Department of Women’s and Children’s Health, Karolinska Institutet and University Hospital, Stockholm, Sweden; 2 Program in Developmental Endocrinology and Genetics, Eunice Kennedy Shriver National Institute of Child Health and Human Development, National Institutes of Health, Bethesda, Maryland, United States of America; 3 Department of Developmental Biology, Faculty of Biology and Centre for Medical Biotechnology, University Duisburg-Essen, Essen, Germany; Stony Brook University, United States of America

## Abstract

Articular and growth plate cartilage are discrete tissues but arise from a common cartilaginous condensation and have comparable spatial architectures consisting of distinct layers of chondrocytes. To investigate similarities and differences between articular and growth plate cartilage and to explore transcriptional changes that occur during the onset of their divergence, we performed manual microdissection of 10-day-old rat proximal tibias, microarray analysis, bioinformatics, and real-time PCR to compare gene expression profiles in individual cartilage layers. We found that many genes that were spatially upregulated in the intermediate/deep zone of articular cartilage were also spatially upregulated in the resting zone of growth plate cartilage (overlap greater than expected by chance, *P*<0.001). Interestingly, the superficial zone of articular cartilage showed an expression profile with similarities to both the proliferative and hypertrophic zones of growth plate cartilage (*P*<0.001 each). Additionally, significant numbers of known proliferative zone markers (3 out of 6) and hypertrophic zone markers (27 out of 126) were spatially upregulated in the superficial zone (more than expected by chance, *P*<0.001 each). In conclusion, we provide evidence that the intermediate/deep zone of articular cartilage has a gene expression profile more similar to that of the resting zone of growth plate cartilage, whereas the superficial zone has a gene expression profile more similar to those of the proliferative and hypertrophic zones. These findings suggest that the superficial zone chondrocytes of articular cartilage differentiate according to a program that is not completely different from but instead has distinct similarities to the hypertrophic differentiation program of growth plate chondrocytes. We also present functional signaling pathways implicated by differential gene expression between articular and growth plate cartilage during their initial separation by the secondary ossification center.

## Introduction

During embryonic development, mesenchymal cells of the mesoderm condense and differentiate into cartilaginous templates at future sites of endochondral bones and become interzone mesenchyme at future sites of articulating joints [1]–[3]. At the start of the fetal period, chondrocytes at the center of the cartilaginous templates stop proliferating, undergo hypertrophy, and release growth factors that attract blood vessels and bone cells, which subsequently form primary ossification centers [4]–[6]. In parallel, interzone mesenchymal cells begin to differentiate into joint capsules, synovial membranes, and ligaments at the periphery and undergo central delimitation giving rise to joint cavities [2], [7]. Around the time of birth, secondary ossification centers form in the middle of the epiphyses at the ends of long bones, compartmentalizing epiphyseal cartilage into articular cartilage peripherally and growth plate cartilage more centrally [8].

Articular and growth plate cartilage thus arise from the same pool of mesenchymal cells. Both tissues can be subdivided into three stratified zones according to chondrocyte size, shape, organization, and function. In growth plate cartilage, the resting zone is located directly beneath the secondary ossification center and contains progenitor cells that continuously renew the proliferative and hypertrophic zones [9]. The underlying proliferative zone contains chondrocytes that replicate at a high rate and line up along the long axis of the bone, forming columns of cells. Farther away from the resting zone, proliferative chondrocytes stop replicating and start enlarging to form the hypertrophic zone. Hypertrophic chondrocytes calcify the extracellular matrix and produce growth factors that attract invading bone cells and blood vessels, which remodel the newly formed cartilage into bone [10]–[12]. In articular cartilage, the superficial zone is exposed to synovial fluid in the joint cavity and contains chondrocytes that are flattened parallel to the articular surface. Cell kinetic studies have indicated that the superficial zone, analogous to the resting zone of growth plate cartilage, contains progenitor cells [13]–[15]. The underlying intermediate zone contains round chondrocytes that tend to orient perpendicular to the articular surface, and farthest from the joint cavity, the deep zone contains larger round chondrocytes that form columns of cells perpendicular to the articular surface [16]–[18]. Although the spatial organization of articular cartilage is similar to that of growth plate cartilage, the tissues have clear functional differences as well as fates and the articular chondrocyte differentiation program is less well characterized [19].

The overall aim of this study was to investigate the transcriptional similarities and differences between articular and growth plate cartilage zones as well as the transcriptional changes that occur during the initial divergence of articular and growth plate cartilage. Based on histological resemblance between growth plate and articular cartilage zones as well as studies indicating that progenitor cells may reside in the superficial zone of articular cartilage, we hypothesized that the gene expression profiles of superficial, intermediate, and deep zones of articular cartilage are similar to those of resting, proliferative, and hypertrophic zones of growth plate cartilage, respectively. Our findings show that there are significant similarities in gene expression patterns between articular and growth plate cartilage, but, in contrast to our hypothesis, demonstrates that the intermediate/deep zone is more similar to the resting zone, whereas the superficial zone is more similar to the proliferative and hypertrophic zones.

## Materials and Methods

### Animal care and handling and ethics statement

Sprague-Dawley rats (Scanbur, Sollentuna, Sweden; Harlan, Indianapolis, IN, USA) were maintained under standardized conditions. 10-day-old rats (n = 8 and n = 4 for microarray and real-time PCR, respectively) were euthanized by carbon dioxide inhalation followed by cervical dislocation. Both proximal tibial epiphyses were rapidly excised, trimmed of any remaining soft connective tissues, bisected sagittally, embedded in Tissue-Tek O.C.T. Compound (Electron Microscopy Sciences, Hatfield, PA, USA), and stored at −80°C until subsequent processing for microdissection. For *in situ* hybridization, proximal tibial epiphyses were fixed in 4% paraformaldehyde (PFA) and decalcified in a solution of 10% ethylenediaminetetraacetic acid (EDTA) and 0.5% PFA. Tissue sections were mounted on Superfrost Plus slides (Histo-Center, Gothenburg, Sweden; Histoserv, Germantown, MD, USA). All animal procedures were approved by the Animal Ethics Committee of Northern Stockholm (Permit number: N290/08) and the Animal Use and Care Committee at the National Institute of Child Health and Human Development (Animal Study Proposal number 11-052).

### Microdissection

**Figure 1:**
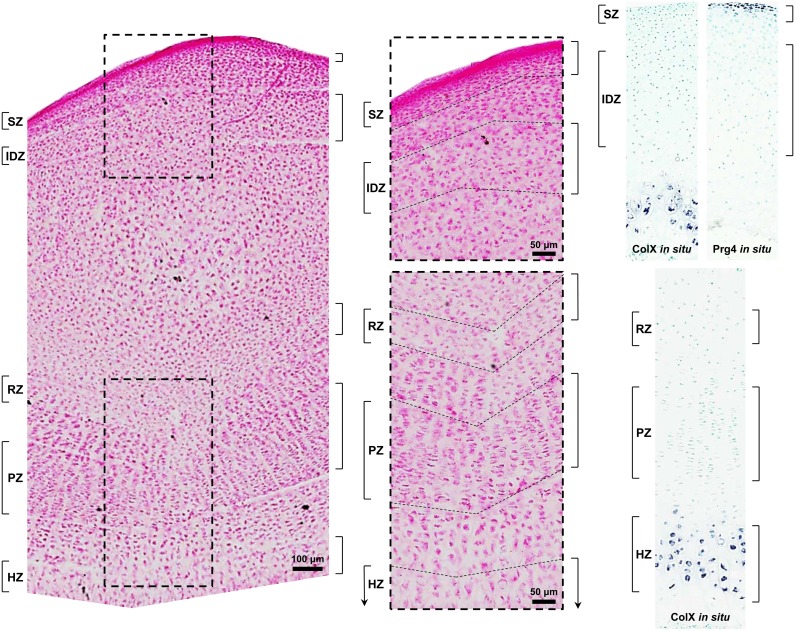
Photomicrograph of a longitudinal section of 10-day-old rat proximal tibia stained with eosin for manual microdissection. Superficial (SZ) and intermediate/deep (IDZ) zones of articular cartilage and resting (RZ), proliferative (PZ), and hypertrophic (HZ) zones of growth plate cartilage were isolated with a razor blade. To minimize cross-contamination, a segment of cartilage between zones was discarded. Higher magnification is shown in the middle panel with respective regions delineated by dashed lines. For microarray analysis, only SZ, IDZ, and RZ were used, whereas all zones were used for real-time PCR. *In situ* hybridization of the articular cartilage SZ marker, *Prg4*, and the hypertrophic chondrocyte marker, *Col10a1* (*ColX*), was used as a visual guide for manual microdissection. Hybridization was detected using NBT/BCIP substrates (purple) and tissues were counterstained with methyl green.

We used 10-day-old animals because, at this age, the secondary ossification center has formed and divides the epiphysis into articular cartilage peripherally and growth plate cartilage more centrally. Manual microdissection was performed as previously described [11] with the following modifications. Sections of proximal tibial epiphyses (60 µm thick) were stained with eosin to visualize histology and dissected using a razor blade under an inverted microscope into superficial zone (SZ), intermediate/deep zone (IDZ), and resting zone (RZ) (Figure 1). *In situ* hybridization for detection of the articular cartilage SZ marker, *Prg4*, and the hypertrophic chondrocyte marker, *Col10a1*, were performed in parallel and provided additional visual guidance for microdissection to localize the superficial zone and avoid the zone of calcification in articular cartilage as well as to localize the hypertrophic zone in growth plate cartilage (Figure 1). SZ was distinguished by high cellularity, small chondrocytes elongated parallel to the articular surface, and high collagen content as determined by strong eosin staining [18], [20]. In order to minimize cross-contamination between SZ and the deeper articular cartilage zones, a layer under the SZ was discarded. In mature articular cartilage, the intermediate and deep zones are histologically distinguished based on chondrocyte size and organization [18]. In young animals, however, the transition from intermediate zone to deep zone can be morphologically indistinguishable [21], [22]. We therefore collected a combined IDZ that contained chondrocytes from both zones, which were distinguished from SZ chondrocytes by their larger size and rounder shape. RZ is located between the primary and secondary ossification centers and was distinguished by chondrocytes, singly or in pairs, that are flat and oriented in the same direction as chondrocytes in the proliferative columns.

### *In situ* hybridization

The gene sequences for rat *Col10a1* and *Prg4* were obtained from the UCSC Genome Browser. Primers were designed using Primer Express 2.0 (Applied Biosystems, Grand Island, NY, USA) and the resulting amplicons were confirmed by NCBI Nucleotide Blast. DNA templates for riboprobe transcription were amplified by PCR using the following reagents: Platinum Taq DNA Polymerase (Invitrogen, Grand Island, NY, USA), cDNA reverse transcribed from total RNA isolated from 3-day-old rat proximal tibial epiphyses, forward primers containing a T7 promoter (5′-TAATACGACTCACTATAGGGAG-3′), and reverse primers containing an Sp6 promoter (5′-TGGATTTAGGTGACACTATAGAAG-3′). Specifically, rat *Col10a1* cDNA (3601–4002 bp of GenBank accession number XM_001053056) forward primer (5′-AAGAGATTTCAGTAAGAGGAGAACAAGG-3′) and reverse primer (5′-TCTGTCCATTCACACCAGGAG-3′) as well as rat *Prg4* cDNA (847–1220 bp and 1593–1943 bp of GenBank accession number NM_001105962) forward primer 1 (5′-TCCCAAGACTACAGCGGCA-3′), reverse primer 1 (5′-GAATGGTGAGTTCAGGCTCCTT-3′), forward primer 2 (5′-CCCCTAAGGAGCCCACATCTAC-3′), and reverse primer 2 (5′-GAGTGGTGGTACTTGCTCTTGGTT-3′) were used. PCR of DNA templates was performed with a 2720 Thermal Cycler (Applied Biosystems) using the following parameters: hold at 94°C for 5 min, followed by 30 cycles of denaturing at 94°C for 30 sec, annealing at 58°C for 30 sec, and extending at 72°C for 45 sec, followed by a final extension at 72°C for 3 min. PCR products were purified by agarose gel electrophoresis and a QIAquick gel extraction kit (Qiagen, Valencia, CA, USA). A second PCR was performed using the same parameters and the products were purified using a QIAquick PCR purification kit (Qiagen). Single stranded riboprobes were transcribed using a digoxigenin (DIG) RNA labeling kit (Roche, Indianapolis, IN, USA) incorporating a DIG-conjugated uracil every 20 to 25 nucleotides. Sp6 polymerase was used for antisense strand riboprobes and T7 polymerase was used for sense strand riboprobes. Riboprobes were purified with Micro Bio-Spin 30 Columns (Bio-Rad, Hercules, CA, USA) and quantified with a NanoDrop Spectrophotometer (Thermo Scientific, Waltham, MA, USA).

Non-radioactive DIG *in* *situ* hybridization was performed as previously described [23] with slight modifications. Briefly, tissue sections (6 µm thick) were baked at 65°C for 1 hr, deparaffinized in xylene, rehydrated through an ethanol series (100%, 100%, 95%, and 70%), and rinsed in PBS. Tissue sections were digested with proteinase K at room temperature for 30 min (100 µg/ml in PBS, pH 7.4), postfixed for 5 min (10% formalin), and acetylated for 15 min (0.25% acetic anhydride in 0.1 M triethanolamine) with each step followed by two 5 min washes in PBS. Prehybridization was carried out at 65°C for 2 hrs in hybridization solution (50% formamide, 10 mM Tris pH 7.6, 200 µg/ml Torula yeast RNA, 1X Denhardt’s solution, 10% dextran sulfate, 600 mM NaCl, 0.25% SDS, 1 mM EDTA, pH 8.0). Hybridization with DIG-labeled riboprobes (100 ng in 100 µl hybridization solution) was performed at 65°C overnight. Posthybridization was carried out by washing with 50% formamide in 1X SSC at 65°C for 30 min, digesting with RNase A (10 µg/ml in 1 M NaCl, 10 mM Tris HCl, 1 mM EDTA, pH 8) at 37°C for 30 min, and washing in SSC at increasing stringency (4X, 1X, 0.5X, and 0.2X). For detection of hybridized riboprobes, tissue sections were rinsed in MABT (0.1 M maleic acid, 0.15 M NaCl, 0.1% v/v Tween-20, pH 7.5), blocked with 1% BSA in MABT at room temperature for 30 min, incubated with alkaline phosphatase-conjugated anti-DIG antibody (Roche) in 1% BSA in MABT at room temperature for 2 hrs, and incubated with nitro blue tetrazolium chloride/5-bromo-4-chloro-3-indolyl phosphate (NBT/BCIP) substrates (Sigma-Aldrich, St. Louis, MO, USA) in NTM (100 mM NaCl, 100 mM Tris pH 9.5, 50 mM MgCl_2_) at room temperature protected from light for 1–3 hrs until a colorimetric change was detected. For mounting, tissue sections were rinsed in PBS for 5 min, fixed in 10% formalin for 20 min, counterstained with methyl green (Vector Laboratories, Burlingame, CA, USA), dehydrated in an ethanol series (70%, 95%, and 100%), cleared in xylene, and mounted with permount. Staining was visualized by scanning the slides under bright field microscopy with a ScanScope CS digital scanner (Aperio Technologies, Inc., Vista, CA, USA).

### RNA isolation

For IDZ and RZ, tissues dissected from both proximal tibias of two animals (42–66 sections) were pooled prior to RNA isolation, and both proximal tibias from a single animal (21–33 sections) were used for SZ. There were 4 samples for each cartilage zone. RNA isolation was performed as previously described [24] except that one-tenth of every volume was used. The final pellet was resuspended in 9 µl diethylpyrocarbonate (DEPC)-treated water. For each animal, 120–800 ng total RNA was extracted from each zone. The RNA integrity numbers were between 7.5 and 8.4 as assessed by a Bioanalyzer 2100 using RNA Pico Chips and version A.02.12 of the Bio Sizing software according to the manufacturer’s instructions (Agilent Biotechnologies, Inc., Palo Alto, CA, USA).

### Microarray analysis

Labeling and hybridization were performed at the Karolinska Institutet Bioinformatics and Expression Analysis core facility, Novum, Huddinge, Sweden (http://www.bea.ki.se/affymetrix/) according to standard Affymetrix protocols. Total RNA (100 ng) extracted from SZ, IDZ, and RZ (n = 4 for each zone) were processed on GeneChip Rat Gene 1.0 ST Arrays (Affymetrix Inc. Santa Clara, CA, USA). Affymetrix CEL files were imported into Partek Genomics Suite 6.6 (Partek Inc., St. Louis, MO) using robust multi-array average analysis, which adjusts for background noise on each array using only the PM probe intensities and subsequently normalizes data across all arrays using quantile normalization followed by median polish summarization to generate a single measure of expression [25], [26]. These expression measures were then log base 2 transformed and lists of spatially regulated genes were generated. A gene was considered spatially regulated if its expression in two adjacent zones differed significantly as determined by a ≥2-fold difference and a one-way ANOVA false discovery rate <0.05. The microarray data have been deposited in NCBI’s Gene Expression Omnibus [27] and are accessible through the GEO Series accession number GSE54216 (http://www.ncbi.nlm.nih.gov/geo/query/acc.cgi?token=ctmhokuctvuhzyf&acc=GSE54216).

### Quantitative real-time PCR

Quantitative real-time PCR was used to confirm microarray gene expression and bioinformatics findings. For this technique, articular cartilage SZ and IDZ as well as growth plate cartilage RZ, PZ, and HZ were manually microdissected from frozen sections of proximal tibial epiphyses of 10-day-old rat (n = 4) as described in the Microdissection section. In addition, PZ chondrocytes were distinguished by the characteristic appearance of the proliferative columns and HZ chondrocytes were distinguished by their larger size. In order to minimize cross-contamination between RZ, PZ, and HZ, layers above and below the PZ were discarded (Figure 1). Total RNA (16–110 ng) was extracted as described in the RNA isolation section and reverse-transcribed into cDNA using Superscript III Reverse Transcriptase (Invitrogen) primed by random hexamers. Intron spanning primers were purchased as prepared assays containing VIC (18S rRNA)- or FAM-labeled TaqMan MGB probes (Applied Biosystems): 18S rRNA (18S-4319413E), Alpl (Rn00564931_m1), Adamts1 (Rn00577887_m1), Mmp9 (Rn00579162_m1), Mmp13 (Rn01448194_m1), Bmp3 (Rn00567346_m1), and Gdf10 (Rn00577682_m1); or designed using Primer Express 2.0 (Applied Biosystems): Prg4 forward (GCATTAACATCCATCCCATGTTT), Prg4 reverse (CCATCCACTGGCTTACCATTG), Col10a1 forward (GCAGCAGCCAGAATCCATTT), Col10a1 reverse (AAGTGCGCTCTTCACACCTGT), Prelp forward (AAGCTGGAACACCTGTACCTCAA), Prelp reverse (GGCAAATCTGGGTCCCATT), Olfml3 forward (ACATGGAACGCCGACTAGCT), Olfml3 reverse (CCGCTTCAGGATCTTCATTGA), Sfrp5 forward (CATCATCGAACATCTCTGTGCAA), and Sfrp5 reverse (CCGTTTTCCTTTTTTACTTCTTTGA). Primers for Grem1 (Rn_Grem1_1_SG) were purchased as a prepared assay (Qiagen). The designed primers were confirmed to generate a single band of the expected size by gel electrophoresis and were validated by dissociation curve analysis. All reactions were run in triplicate using TaqMan Universal PCR Master Mix or SYBR Green Master Mix (Applied Biosystems) with the ViiA7 Real-Time PCR System (Applied Biosystems) set at the following thermal cycling condition: one cycle at 50°C for 2 min and 95°C for 10 min, followed by 40 cycles of 15 s at 95°C and 1 min at 60°C. In order to account for variability in the initial concentration and quality of total RNA, the relative amounts of transcripts were normalized to the housekeeping gene 18S ribosomal RNA. Relative expression was calculated by the delta-delta C_T_ method using the formula: Relative Expression_i_ = 2^–(CT,i – CT,18S)^×10^6^, where i represents the gene of interest and C_T_ represents the threshold cycle. Relative expression values were multiplied by 10^6^ to produce more convenient numbers.

### Bioinformatics and statistical analysis

Comparison of microarray gene expression levels was performed by one-way ANOVA using log base 2 transformed relative expression data (Partek Genomics Suite 6.6). All *P*-values were two-tailed and significance was recognized at a *P*-value corresponding to a false discovery rate <0.05. Principal components analysis on all genes followed by unsupervised hierarchical cluster analysis and heat map visualization on genes differentially expressed between SZ and IDZ were used to assess whether the gene expression profile of SZ or IDZ of articular cartilage is more similar to that of growth plate cartilage RZ (Partek Genomics Suite 6.6). To compare spatial gene expression of articular cartilage to all three zones of growth plate cartilage, we combined the current microarray dataset with our previously published microarray dataset of resting (RZ), proliferative (PZ), and hypertrophic (HZ) zones of growth plate cartilage from 7-day-old Sprague-Dawley rats [11], [28]. For this analysis, we assumed that gene expression patterns of individual growth plate cartilage zones in 7- and 10-day old rats are similar since the morphology and organization of individual zones are similar and we have previously shown that the genes that change with zone are mostly different from those that change with age [28]. We identified 12,593 genes that were present on both microarray platforms (Microsoft Excel 2010). To avoid selection bias, all possible comparisons between the spatially upregulated genes of growth plate cartilage zones were made with those of articular cartilage zones. The probability of overlapping genes occurring by chance between zones across microarray datasets was determined using Pearson’s chi-square test and correction for multiple comparisons was performed using the Holm-Sidak method (SigmaPlot 10). Finally, expression levels of known growth plate cartilage zonal markers [28] were assessed in SZ and IDZ of articular cartilage. Of the published markers, 37 RZ, 6 PZ, and 126 HZ markers were present on the current microarray platform, and the significance of their overlaps with spatially upregulated genes in SZ and IDZ were determined using Pearson’s chi-square test (SigmaPlot 10). For real-time PCR data, statistical analysis was performed on log base 2 transformed relative expression data using repeated measures ANOVA to assure significant differences in means between zones followed by paired t-test to make the predetermined comparisons of SZ to IDZ, RZ to PZ, PZ to HZ, and RZ to HZ (SigmaPlot 10). All *P*-values were two-tailed and significance was recognized at *P*<0.05.

## Results

To compare transcriptional patterns between articular and growth plate cartilage, we microdissected rat proximal tibial epiphyses and collected the superficial and intermediate/deep zones from articular cartilage and the resting zone from growth plate cartilage. We then used bioinformatic approaches to define gene expression similarities and differences between articular and growth plate cartilage zones. In addition, we combined these data with our previous expression data from individual zones of growth plate cartilage to further study the similarities and differences in gene expression between articular and growth plate cartilage. We also confirmed 12 selected genes from our microarray and bioinformatic analyses by microdissecting articular and growth plate cartilage from a new set of animals and assessing gene expression by real-time PCR. Lastly, we performed Ingenuity Pathways Analysis (Ingenuity Systems, www.ingenuity.com) on overlapping gene expression between articular and growth plate cartilage zones as well as on differential gene expression in articular versus growth plate cartilage during their initial separation by the secondary ossification center.

### The intermediate/deep zone, not the superficial zone, of articular cartilage shows transcriptional similarities to the resting zone of growth plate cartilage

**Figure 2:**
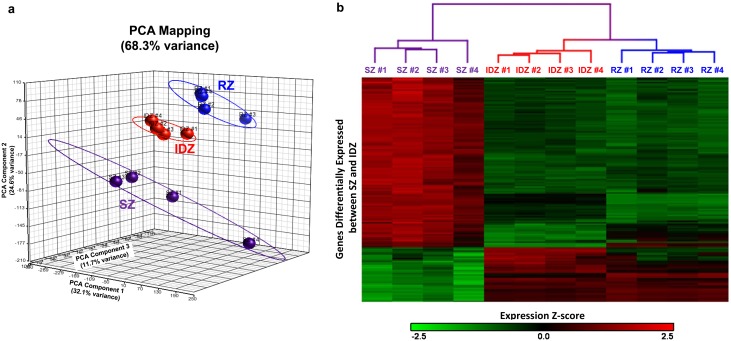
Bioinformatic comparison of articular and growth plate cartilage zones. Bioinformatics was performed on microarray gene expression of SZ and IDZ of articular cartilage and RZ of growth plate cartilage. (**a**) Principal components analysis scatter plot in three dimensions retaining 68.3% of original sample variation. (**b**) Dendrogram following unsupervised hierarchical cluster analysis organizing samples by similarity and heat map visualization of gene expression using only genes differentially expressed between SZ and IDZ. Red corresponds to higher gene expression levels represented by z-score. SZ, superficial zone; IDZ, intermediate/deep zone; RZ, resting zone.

We first compared superficial (SZ), intermediate/deep (IDZ), and resting (RZ) zones using principal components analysis and found that the samples of each individual zone grouped together, thus validating the accuracy of our manual microdissection (Figure 2a). In contrast to our hypothesis, this analysis indicated that RZ of growth plate cartilage was more similar to IDZ than SZ of articular cartilage as determined by closer proximity of respective spheres (Figure 2a). We then used unsupervised hierarchical cluster analysis to compare zonal expression of genes differentially expressed between SZ and IDZ (≥2-fold, false discovery rate <0.05, Table 1 and Table S1 in File S1) and found that the RZ samples cluster more closely with the IDZ samples than with the SZ samples (Figure 2b), thus again indicating that the gene expression profile of articular cartilage IDZ, not SZ, is more similar to that of growth plate cartilage RZ. Next, we visualized the expression of the same differentially expressed genes in RZ using heat map visualization. Genes that were upregulated in IDZ compared to SZ tended to be highly expressed in RZ, whereas genes that were upregulated in SZ compared to IDZ tended to be expressed at lower levels in RZ (Figure 2b).

**Table 1:** Top differentially expressed genes between superficial and intermediate/deep zones of articular cartilage.

Gene Symbol	Superficial ZoneRaw Signal	Intermediate/DeepZone Raw Signal	Fold-Change		*P*-value
*Tnmd*	940	39	24	Up in SZ	8.66E-10
*Aspn*	3164	131	24	Up in SZ	9.13E-07
*Kera*	1100	54	20	Up in SZ	7.61E-09
*Rgs5*	3077	206	15	Up in SZ	2.03E-07
*Cd34*	2308	157	15	Up in SZ	6.85E-07
*Tppp3*	2303	158	15	Up in SZ	5.20E-08
*Igf1*	2789	211	13	Up in SZ	2.96E-07
*Dpt*	2086	171	12	Up in SZ	1.14E-06
*Cd55*	714	61	12	Up in SZ	8.32E-06
*Thbs4*	2187	198	11	Up in SZ	1.08E-08
*Olr63*	1202	109	11	Up in SZ	3.44E-09
*Dkk3*	1548	141	11	Up in SZ	2.13E-07
*Olfml1*	444	46	10	Up in SZ	7.18E-08
*Egfl6*	597	63	10	Up in SZ	3.55E-07
*Fndc1*	2475	264	9	Up in SZ	1.10E-07
*Gpr116*	555	59	9	Up in SZ	8.66E-08
*Ednrb*	559	60	9	Up in SZ	1.20E-08
*Sepp1*	1252	137	9	Up in SZ	5.30E-08
*Tinagl1*	1029	115	9	Up in SZ	2.67E-06
*Lgi1*	220	25	9	Up in SZ	1.40E-05
*Art3*	77	669	9	Up in IDZ	1.09E-10
*Moxd1*	171	1200	7	Up in IDZ	8.96E-08
*Efemp1*	133	912	7	Up in IDZ	7.88E-10
*Epyc*	291	1964	7	Up in IDZ	2.88E-05
*Ctxn3*	120	796	7	Up in IDZ	1.18E-08
*Rspo3*	212	1344	6	Up in IDZ	8.29E-09
*Cntn1*	129	721	6	Up in IDZ	1.26E-07
*Fam151a*	87	466	5	Up in IDZ	4.82E-09
*Grem1*	217	1100	5	Up in IDZ	3.13E-06
*Bmp3*	186	919	5	Up in IDZ	1.27E-06
*Car12*	129	625	5	Up in IDZ	7.01E-08
*A2m*	151	720	5	Up in IDZ	2.73E-07
*Unc5c*	269	1264	5	Up in IDZ	1.99E-07
LOC685203	156	716	5	Up in IDZ	4.84E-05
*Sfrp1*	189	853	5	Up in IDZ	1.04E-05
*Ninj2*	183	794	4	Up in IDZ	1.28E-05
*Lrrn3*	96	404	4	Up in IDZ	1.22E-05
*A1i3*	102	416	4	Up in IDZ	4.68E-06
*Fras1*	61	243	4	Up in IDZ	9.34E-09
*Pla2g5*	213	855	4	Up in IDZ	3.42E-07

*SZ, superficial zone; IDZ, intermediate/deep zone.

### The transition from intermediate/deep zone to superficial zone exhibits transcriptional similarities with the growth plate chondrocyte differentiation program

**Figure 3:**
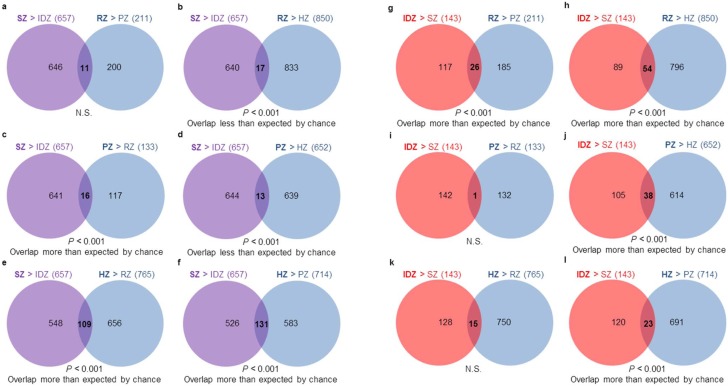
Number of spatially upregulated genes that overlap between articular and growth plate cartilage zones. Pearson’s chi-square test was used to determine whether an overlap is statistically significant. Significance indicates that an overlap is more or less than expected by chance, whereas non-significance indicates that an overlap is what would be expected by chance. Based on overlaps, SZ is more similar to HZ than other growth plate cartilage zones (**b, d–f**) and more similar to PZ than RZ (**a, c**), whereas IDZ is more similar to RZ than other growth plate cartilage zones (**g–i, k**) and has similarities to both PZ and HZ (**j, l**). SZ, superficial zone; IDZ, intermediate/deep zone; RZ, resting zone; PZ, proliferative zone; HZ, hypertrophic zone; N.S., not significant.

**Figure 4:**
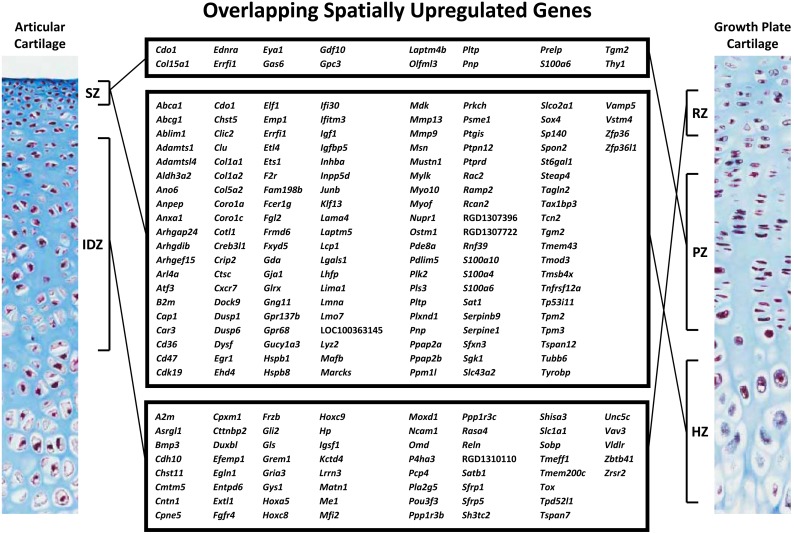
Spatially upregulated genes that overlap between articular and growth plate cartilage zones. SZ, superficial zone; IDZ, intermediate/deep zone; RZ, resting zone; PZ, proliferative zone; HZ, hypertrophic zone. Left and right panels are photomicrographs of 10-day-old rat articular and growth plate cartilage, respectively, stained with Masson’s Trichrome. Colors: black, nuclei; blue, collagen; red, cytoplasm.

We next compared gene expression changes between SZ and IDZ of the current study that used 10-day-old rat proximal tibias with those occurring between growth plate cartilage resting (RZ), proliferative (PZ), and hypertrophic (HZ) zones from a previous microarray dataset using proximal tibial epiphyses of 7-day-old rats [11], [25]. Since the current articular zonal expression data were generated on a different microarray platform (Rat Gene 1.0 ST) than the previous growth plate cartilage expression data (Rat Genome 230 2.0), expression levels could not be directly compared between articular and growth plate cartilage zones. Instead, we used Pearson’s chi-square tests to assess whether the overlaps of spatially upregulated genes between articular and growth plate cartilage zones were significant or not. All possible comparisons between spatially regulated genes of growth plate cartilage zones were made with those of articular cartilage zones. First, focusing on SZ, we found that many genes that were expressed at higher levels in SZ versus IDZ of articular cartilage were also expressed at higher levels in PZ versus RZ, HZ versus RZ, and HZ versus PZ of growth plate cartilage (overlaps greater than expected by chance, *P*<0.001 each, Figure 3c, 3e, and 3f, respectively). Conversely, the genes that were upregulated in SZ versus IDZ were not overrepresented among the genes upregulated in RZ versus PZ (N.S., Figure 3a), RZ versus HZ, and PZ versus HZ (overlaps less than expected by chance, *P*<0.001, Figure 3b and 3d, respectively). These findings show that the gene expression profile of articular cartilage SZ is more similar to growth plate cartilage PZ and HZ than to RZ, suggesting that the transition from IDZ to SZ has transcriptional similarities with the growth plate chondrocyte differentiation program. Next focusing on IDZ, we found that a significant number of genes that were spatially upregulated in IDZ versus SZ were also upregulated in RZ versus PZ, RZ versus HZ, and PZ versus HZ (overlaps greater than expected by chance, *P*<0.001 each, Figure 3g, 3h, 3j, respectively). Conversely, genes that were upregulated in IDZ versus SZ were not enriched in PZ versus RZ or HZ versus RZ (N.S. each, Figure 3i and 3k, respectively). These findings show that the gene expression profile of articular cartilage IDZ has closer resemblance to growth plate cartilage RZ and PZ than to HZ, suggesting again that the transition from IDZ to SZ has transcriptional similarities with the growth plate chondrocyte differentiation program. Interestingly, there was also a significant overlap of spatially upregulated genes between IDZ versus SZ and HZ versus PZ (overlap more than expected by chance, *P*<0.001, Figure 3l). This overlap thus identified genes that were enriched during hypertrophic differentiation of growth plate cartilage but downregulated in the transition from IDZ to SZ of articular cartilage. Ingenuity Pathways Analysis on the spatially upregulated genes that overlap significantly between articular and growth plate cartilage zones (Figure 4) implicated biologically relevant pathways in articular cartilage SZ and growth plate cartilage HZ as well as in articular cartilage IDZ and growth plate cartilage RZ (Table 2 and Table S2 in File S1).

**Table 2:** Top signaling pathways implicated by spatially upregulated genes that overlap between superficial and hypertrophic zones as well as intermediate/deep and resting zones.

Zones	Name	Ratio	*P*-value	Regulated Genes
SZ and HZ	Inhibition of Angiogenesisby TSP1	4/31 (13%)	1.58E-04	*CD47,GUCY1A3,CD36,MMP9*
	Hepatic Fibrosis/HepaticStellate Cell Activation	7/133 (5%)	1.70E-04	*COL1A2,COL1A1,IGF1,MMP13,* *IGFBP5,SERPINE1,MMP9*
	Atherosclerosis Signaling	6/111 (5%)	3.55E-04	*COL1A2,COL1A1,CD36,MMP13,* *MMP9, TNFRSF12A*
	Axonal Guidance Signaling	11/406 (3%)	7.41E-04	*RAC2,GNG11,TUBB6,IGF1,ADAMTS1,* *ARHGEF1,MMP13,PRKCH,* *PLXND1,ABLIM1,MMP9*
	ERK/MAPK Signaling	7/179 (4%)	9.55E-04	*ETS1,RAC2,DUSP1,DUSP6,* *PPM1L,HSPB1,ELF1*
	Natural Killer Cell Signaling	5/93 (5%)	1.45E-03	*RAC2,TYROBP,FCER1G,PRKCH,* *INPP5D*
	LXR/RXR Activation	5/107 (5%)	2.29E-03	*CD36,ABCG1,PLTP,MMP9,* *ABCA1*
	Guanosine NucleotidesDegradation III	2/12 (17%)	4.90E-03	*GDA,PNP*
	Xanthine and Xanthosine Salvage	1/1 (100%)	8.91E-03	*PNP*
	Dendritic Cell Maturation	5/155 (3%)	9.77E-03	*B2M,COL1A2,COL1A1,TYROBP,* *FCER1G*
IDZ and RZ	Glutamate Receptor Signaling	3/59 (5%)	8.71E-04	*GLS,SLC1A1,GRIA3*
	Role of Osteoblasts,Osteoclasts and Chondrocytesin Rheumatoid Arthritis	4/215 (2%)	6.03E-03	*FRZB,BMP3,SFRP5,SFRP1*
	Asparagine Degradation I	1/2 (50%)	6.92E-03	*ASRGL1*
	Glutamine Degradation I	1/2 (50%)	6.92E-03	*GLS*
	Heparan SulfateBiosynthesis (Late Stages)	2/43 (5%)	9.77E-03	*CHST11,EXTL1*
	Heparan SulfateBiosynthesis	2/50 (4%)	1.32E-02	*CHST11,EXTL1*
	Glycogen Biosynthesis II(from UDP-D-Glucose)	1/5 (20%)	1.74E-02	*GYS1*
	Wnt/β-cateninSignaling	3/158 (2%)	1.78E-02	*FRZB,SFRP5,SFRP1*
	Basal Cell CarcinomaSignaling	2/66 (3%)	2.14E-02	*GLI2,BMP3*
	BMP signaling pathway	2/74 (3%)	2.63E-02	*BMP3,GREM1*

*SZ, superficial zone; IDZ, intermediate/deep zone; RZ, resting zone; HZ, hypertrophic zone.

We subsequently assessed expression levels of known growth plate cartilage zonal gene markers [28] in SZ and IDZ of articular cartilage. Similar to the previous patterns, resting zone markers were significantly overrepresented (9 out of 37) in the list of genes upregulated in IDZ compared to SZ (more than expected by chance, *P*<0.001, Table 3). Interestingly, there was also a significant overrepresentation of resting zone markers (5 out of 37) in the list of genes upregulated in SZ compared to IDZ (more than expected by chance, *P*<0.001, Table 3). A significant proportion of proliferative zone markers (3 out of 6) were found to be significantly upregulated in SZ compared to IDZ (more than expected by chance, *P*<0.001, Table 3), whereas none were upregulated in IDZ compared to SZ (N.S., Table 3). Also similar to the previous patterns, 27 out of 126 hypertrophic zone markers were upregulated in SZ compared to IDZ (more than expected by chance, *P*<0.001, Table 3), whereas only 3 of 126 were upregulated in IDZ compared to SZ (N.S., Table 3). These findings indicate that both SZ and IDZ have transcriptional similarities with RZ, but that only SZ has a transcriptional profile similar to PZ and HZ.

**Table 3:** Expression of growth plate cartilage zonal markers in superficial and intermediate/deep zones of articular cartilage.

Resting Zone Markers					
Gene Symbol	Superficial ZoneRaw Signal(Upregulated markers: 5)	Intermediate/Deep ZoneRaw Signal(Upregulated markers: 9)	Fold-Change		*P*-value
*Ptn*	2268	538	4	Up in SZ	1.09E-07
*Hhip*	363	92	4	Up in SZ	2.19E-07
*Col14a1*	5864	1596	4	Up in SZ	2.80E-11
RGD1562717	3695	1257	3	Up in SZ	3.09E-06
*Pdgfra*	1280	606	2	Up in SZ	2.78E-06
*Efemp1*	133	912	7	Up in IDZ	7.88E-10
*Grem1*	217	1100	5	Up in IDZ	3.13E-06
*Bmp3*	186	919	5	Up in IDZ	1.27E-06
*Lrrn3*	96	404	4	Up in IDZ	1.22E-05
*Shisa3*	310	1196	4	Up in IDZ	1.15E-08
*Sfrp5*	367	1302	4	Up in IDZ	4.91E-06
*Pou3f3*	438	1504	3	Up in IDZ	2.89E-08
*Pcp4*	1148	3930	3	Up in IDZ	1.98E-07
*Ppp1r3b*	250	832	3	Up in IDZ	7.02E-08
**Proliferative Zone Markers**					
**Gene Symbol**	**Superficial Zone** **Raw Signal** **(Upregulated markers: 3)**	**Intermediate/Deep** **Zone Raw Signal** **(Upregulated markers: 0)**	**Fold-Change**		***P*** **-value**
*Gdf10*	484	184	3	Up in SZ	8.24E-05
*Olfml3*	2403	941	3	Up in SZ	6.34E-08
*Prelp*	3664	1463	3	Up in SZ	3.39E-07
**Hypertrophic Zone Markers**					
**Gene Symbol**	**Superficial Zone** **Raw Signal** **(Upregulated markers: 27)**	**Intermediate/Deep Zone** **Raw Signal** **(Upregulated markers: 3)**	**Fold-Change**		***P*** **-value**
RGD1307396	542	90	6	Up in SZ	3.93E-06
*Fxyd5*	1358	274	5	Up in SZ	4.10E-06
*Col5a2*	1226	249	5	Up in SZ	1.51E-06
*Mmp13*	3448	705	5	Up in SZ	4.98E-03
*Tmsb4x*	376	78	5	Up in SZ	2.14E-06
*Ctsk*	1561	337	5	Up in SZ	4.09E-04
*Mafb*	1175	255	5	Up in SZ	1.98E-06
*Mmp9*	3761	824	5	Up in SZ	2.48E-03
*Spon2*	1041	247	4	Up in SZ	4.94E-06
*Col1a1*	13700	3598	4	Up in SZ	7.23E-04
*Col1a2*	11867	3128	4	Up in SZ	2.66E-04
*B2m*	4002	1097	4	Up in SZ	1.38E-07
*Inhba*	410	118	3	Up in SZ	5.10E-06
*Adamts1*	405	118	3	Up in SZ	3.19E-06
*Steap4*	625	189	3	Up in SZ	2.02E-05
*Anpep*	703	213	3	Up in SZ	1.85E-05
*Tgm2*	747	229	3	Up in SZ	1.45E-05
*Serpine1*	312	99	3	Up in SZ	3.40E-05
*Fgl2*	212	75	3	Up in SZ	4.35E-06
*Emp1*	3186	1148	3	Up in SZ	3.99E-07
*Hspb1*	475	178	3	Up in SZ	2.62E-06
*Pdlim5*	627	246	3	Up in SZ	2.90E-09
*S100a6*	453	195	2	Up in SZ	1.28E-04
*Tnfrsf12a*	472	205	2	Up in SZ	1.98E-06
*Ibsp*	262	123	2	Up in SZ	5.42E-02
*Ptgis*	492	233	2	Up in SZ	2.65E-05
*Rac2*	334	165	2	Up in SZ	9.58E-03
*Alox12*	64	197	3	Up in IDZ	6.22E-07
*Sgms2*	289	881	3	Up in IDZ	7.12E-06
*Gmpr*	184	382	2	Up in IDZ	1.08E-05

*SZ, superficial zone; IDZ, intermediate/deep zone.

### Validation of microdissection and microarray analysis

**Figure 5:**
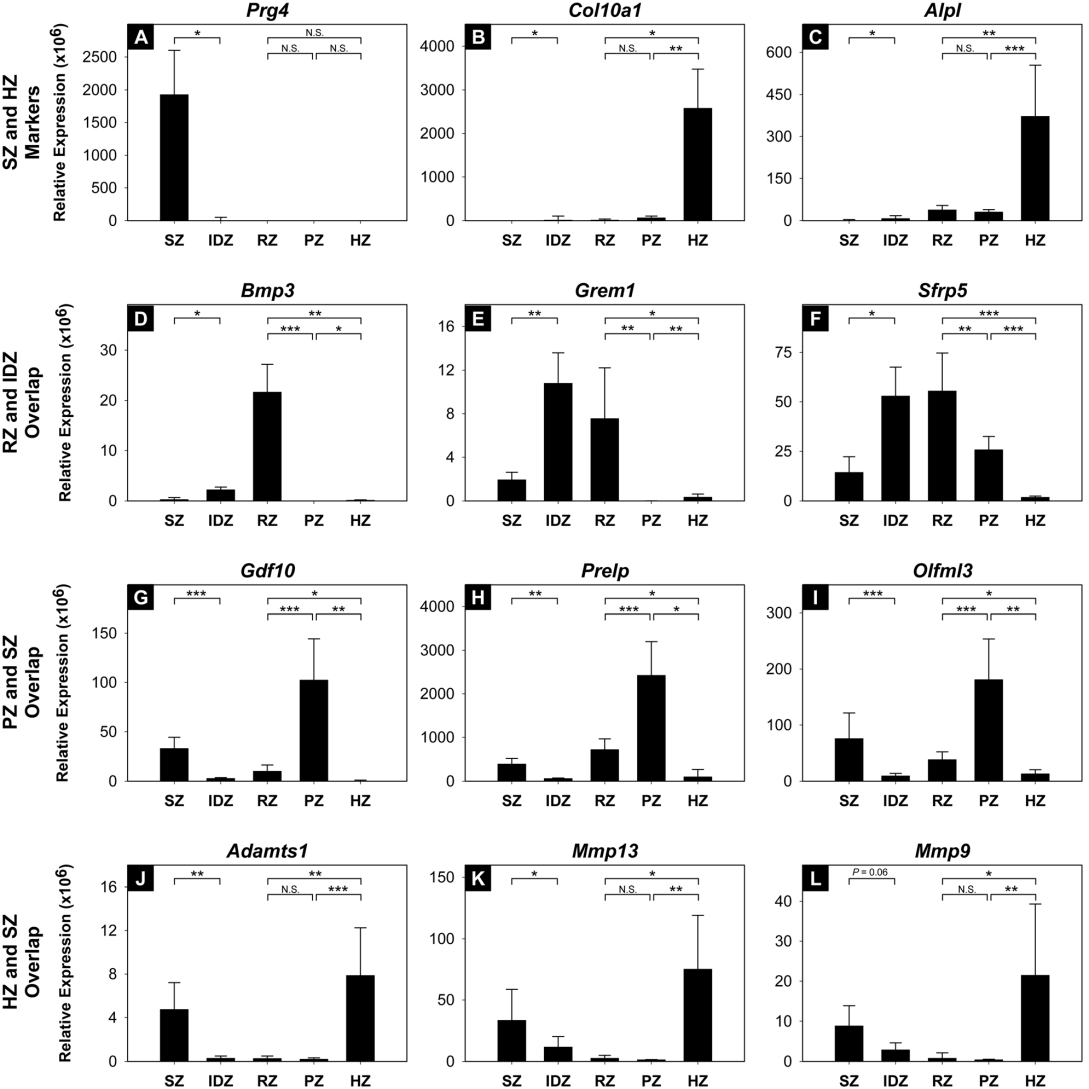
Real-time PCR of spatially upregulated genes that overlap between articular and growth plate cartilage zones. Relative expression of selected genes that overlap between RZ and IDZ, including *Bmp3*, *Grem1*, and *Sfrp5* (**D–F**), PZ and SZ, including *Gdf10*, *Prelp*, and *Olfml3* (**G–I**), as well as HZ and SZ, including *Adamts1*, *Mmp13*, and *Mmp9* (**J–L**), was determined in proximal tibial epiphyses of 10-day-old rats (n = 4) and values were normalized to 18S rRNA. Accuracy of the microdissection technique was validated by inspecting relative expression of *Prg4*, *Col10a1*, and *Alpl* (**A–C**). SZ, superficial zone; IDZ, intermediate/deep zone; RZ, resting zone; PZ, proliferative zone; HZ, hypertrophic zone; N.S., not significant; *P<0.05; **P<0.01; ***P<0.001.

In order to validate the accuracy of the microdissection technique (Figure 1) as well as to test the assumption that gene expression of individual growth plate cartilage zones are similar in 7 and 10 day-old rats, we microdissected articular cartilage SZ and IDZ as well as growth plate cartilage RZ, PZ, and HZ from 10-day-old rats (n = 4) and studied 12 selected genes by real-time PCR. For this validation experiment, one well established SZ marker (*Prg4*; Figure 5A) and two HZ markers (*Col10a1, Alpl;* Figure 5B and 5C) as well as genes found to be spatially upregulated in both RZ and IDZ (*Bmp3*, *Grem1*, *Sfrp5;* Figure 4 & 5D–5F), PZ and SZ (*Gdf10*, *Olfml3*, *Prelp;* Figure 4 & 5G–5I), and HZ and SZ (*Adamts1*, *Mmp13*, *Mmp9,* Figure 4 & 5J–5L) were selected. We found that *Prg4* was expressed at least 200-fold higher in SZ versus all other zones (*P*<0.05; Figure 5A), whereas *Col10a1* and *Alpl* were expressed at least 40-fold (*P*<0.05 for all comparisons) and 12-fold higher (*P*<0.05 for all comparisons), respectively, in HZ compared to all other zones (Figure 5B and 5C). Furthermore, all of the selected genes showed expression patterns similar to those found using microarray analysis (Figure 5A–5L). In other words, zonal gene expression found to be significantly different by microarray analysis was also found to be significantly different in the new set of samples assessed by real-time PCR, with the only exception being *Mmp9.* Similar to the microarray analysis, *Mmp9* was found to be significantly upregulated in HZ compared to PZ and RZ of growth plate cartilage and also showed the expected trend of higher expression levels in articular SZ compared to IDZ (*P* = 0.06; Figure 5L); however, the *P*-value was larger than the preset requirement for significance of *P*<0.05.

### Differential gene expression between the intermediate/deep zone of articular cartilage and the resting zone of growth plate cartilage

IDZ and RZ originate from the same pool of chondrocytes but are physically separated by the secondary ossification center starting at approximately postnatal day 7. In order to characterize early gene expression changes that differentiate articular and growth plate cartilage, we identified genes that were differentially expressed between IDZ and RZ in 10-day-old rat proximal tibial epiphyses (≥2-fold, false discovery rate <0.05, Table 4 and Table S3 in File S1). Genes highly expressed in articular cartilage IDZ compared to growth plate cartilage RZ included articular cartilage marker *Prg4* as well as periostin (*Postn*) and Wnt inhibitory factor 1 (*Wif1*), whereas genes highly expressed in growth plate cartilage RZ compared to articular cartilage IDZ included hedgehog interacting protein (*Hhip*), BMP signaling inhibitor *Bmp3*, and RZ marker *Sfrp5*, which is also an inhibitor of Wnt signaling. Ingenuity Pathways Analysis implicated biologically relevant pathways in the gene expression difference between IDZ and RZ, including Role of Osteoblasts, Osteoclasts and Chondrocytes in Rheumatoid Arthritis that was more active in IDZ as well as sonic hedgehog (SHH) and bone morphogenetic protein (BMP) signaling pathways that were more active in RZ (Table 5 and Table S4 in File S1).

**Table 4:** Top differentially expressed genes between intermediate/deep zone of articular cartilage and resting zone of growth plate cartilage.

Gene Symbol	Intermediate/DeepZone Raw Signal	Resting ZoneRaw Signal	Fold-Change		*P*-value
*Prg4*	1265	51	25	Up in IDZ	3.07E-06
*Fap*	342	24	14	Up in IDZ	4.73E-11
*Gnas*	803	121	7	Up in IDZ	2.83E-08
*Cytl1*	624	97	6	Up in IDZ	1.55E-07
*Postn*	427	78	6	Up in IDZ	7.61E-07
*Kcnt2*	223	44	5	Up in IDZ	3.78E-09
*Sorcs3*	227	50	5	Up in IDZ	1.36E-07
LOC314942	944	211	4	Up in IDZ	4.04E-05
*Sim2*	334	76	4	Up in IDZ	1.51E-08
*Col3a1*	973	249	4	Up in IDZ	5.53E-07
*Vcan*	350	90	4	Up in IDZ	2.99E-06
*Rhoj*	671	182	4	Up in IDZ	3.33E-07
*Tbx5*	194	53	4	Up in IDZ	7.94E-04
*Sulf2*	1814	506	4	Up in IDZ	1.11E-06
*Etv1*	387	109	4	Up in IDZ	7.38E-07
*Enpep*	465	131	4	Up in IDZ	4.33E-06
*Cfh*	398	113	4	Up in IDZ	1.11E-05
*Dpt*	171	50	3	Up in IDZ	3.19E-04
*Fam151a*	466	142	3	Up in IDZ	1.03E-07
*Agtr2*	367	113	3	Up in IDZ	1.68E-06
*Hhip*	92	1195	13	Up in RZ	9.13E-10
LOC680415	145	1085	7	Up in RZ	4.87E-08
*F13a1*	244	1730	7	Up in RZ	1.74E-06
*Tnni2*	361	2433	7	Up in RZ	1.18E-07
*Ntn1*	211	1334	6	Up in RZ	2.27E-08
*Car8*	83	470	6	Up in RZ	1.24E-08
*Casq1*	64	340	5	Up in RZ	6.24E-08
*Fa2h*	51	270	5	Up in RZ	3.37E-07
*Slc40a1*	319	1583	5	Up in RZ	1.98E-08
*Cadm2*	148	720	5	Up in RZ	3.44E-08
*Mfap3l*	90	427	5	Up in RZ	2.67E-06
*Il17b*	365	1676	5	Up in RZ	1.71E-07
*Bmp7*	277	1188	4	Up in RZ	4.15E-06
*Serpine2*	380	1576	4	Up in RZ	4.13E-07
*Kcnip1*	66	272	4	Up in RZ	1.07E-06
*Cpa6*	53	195	4	Up in RZ	1.09E-08
*Nptx1*	131	467	4	Up in RZ	3.93E-06
*Sfrp5*	1302	4615	4	Up in RZ	4.91E-06
*Cxcr4*	140	491	4	Up in RZ	6.36E-08
*Bai1*	184	637	3	Up in RZ	3.41E-06

*IDZ, intermediate/deep zone; RZ, resting zone.

**Table 5:** Top signaling pathways implicated by differentially expressed genes between intermediate/deep zone of articular cartilage and resting zone of growth plate cartilage.

Zone	Name	Ratio	*P*-value	Regulated Genes
Up in IDZ	Role of Osteoblasts,Osteoclasts and Chondrocytesin Rheumatoid Arthritis	6/215 (3%)	3.80E-03	*WIF1,FZD4,IGF1,MMP3,DLX5,* *BMP5*
	IGF-1 Signaling	4/97 (4%)	4.68E-03	*NOV,IGF1,IGFBP5,IGFBP7*
	Growth Hormone Signaling	3/67 (4%)	1.15E-02	*GHR,IGF1,A2M*
	Hepatic Fibrosis/HepaticStellate Cell Activation	4/133 (3%)	1.41E-02	*IGF1,IGFBP5,A2M,COL3A1*
	LPS/IL-1 Mediated Inhibitionof RXR Function	5/214 (2%)	1.48E-02	*GSTM1,APOE,SLC27A6,HS6ST2,* *SOD3*
	Factors PromotingCardiogenesis in Vertebrates	3/88 (3%)	2.29E-02	*TBX5,FZD4,BMP5*
	Oncostatin M Signaling	2/33 (6%)	2.29E-02	*EPAS1,MMP3*
	Inhibition of MatrixMetalloproteases	2/36 (6%)	2.69E-02	*MMP3,A2M*
	Atherosclerosis Signaling	3/111 (3%)	3.89E-02	*APOE,MMP3,COL3A1*
	Superoxide RadicalsDegradation	1/6 (17%)	4.17E-02	*SOD3*
Up in RZ	GADD45 Signaling	4/19 (21%)	2.82E-06	*PCNA,CCNE1,CCND1,CCNB1*
	Sonic Hedgehog Signaling	4/25 (16%)	1.41E-05	*PRKAR2B,PTCH1,HHIP,CCNB1*
	Basal Cell CarcinomaSignaling	5/66 (8%)	4.47E-05	*BMP3,PTCH1,LEF1,BMP7,HHIP*
	Mitotic Roles of Polo-LikeKinase	4/61 (7%)	4.68E-04	*CDC20,PTTG1,PLK1,CCNB1*
	Axonal Guidance Signaling	9/406 (2%)	5.75E-04	*PRKAR2B,LOC680415,MAG,CXCR4,* *BMP3,PTCH1,BMP7,HHIP,NTN1*
	Molecular Mechanisms ofCancer	8/330 (2%)	5.75E-04	*CCNE1,PRKAR2B,BMP3,* *PTCH1,LEF1, BMP7,CCND1,CASP7*
	BMP signaling pathway	4/74 (5%)	9.33E-04	*PRKAR2B,RUNX2,BMP3,BMP7*
	p53 Signaling	4/82 (5%)	1.51E-03	*PCNA,BAI1,CCND1,SERPINE2*
	Role of Osteoblasts,Osteoclasts and Chondrocytesin Rheumatoid Arthritis	6/215 (3%)	1.66E-03	*RUNX2,BMP3,SFRP5,LEF1,BMP7,ALPL*
	Factors PromotingCardiogenesis in Vertebrates	4/88 (5%)	1.74E-03	*CCNE1,BMP3,LEF1,BMP7*

*IDZ, intermediate/deep zone; RZ, resting zone.

## Discussion

In the present study, we used manual microdissection and gene expression microarray analysis followed by real-time PCR of selected genes to characterize spatial gene expression profiles of articular and growth plate cartilage zones. First, we identified differential gene expression in articular cartilage superficial (SZ) and intermediate/deep (IDZ) zones and used bioinformatic approaches to compare the expression patterns in articular cartilage with growth plate cartilage resting zone (RZ) and found that RZ had a gene expression profile more similar to IDZ than SZ. We then compared differentially expressed genes in SZ and IDZ of articular cartilage with a previous gene expression dataset of individual growth plate cartilage zones and again found that there was a significant overlap in upregulated genes between IDZ and RZ as well as between SZ and growth plate cartilage proliferative (PZ) and hypertrophic (HZ) zones. Next, we identified functional pathways implicated by the overlapping gene expression patterns of articular and growth plate cartilage zones as well as functional pathways implicated in the early differentiation of articular versus growth plate cartilage. Finally, we assessed the expression levels of growth plate cartilage zonal markers in SZ and IDZ and again found that gene expression patterns of IDZ resembled RZ, whereas SZ resembled PZ and HZ.

There were some technical limitations in this study. First, since it is difficult to histologically distinguish between the intermediate and deep zones of articular cartilage in 10-day-old rats, we could not collect the zones separately using manual microdissection. This decreased the number of comparisons we could make between articular and growth plate cartilage zones and, in turn, decreased the resolution of our findings. Second, the current articular cartilage and previous growth plate cartilage datasets were obtained from different experiments and microarray platforms. Therefore, besides comparing articular cartilage SZ and IDZ to growth plate cartilage RZ directly since they were processed on the same microarray platform, we could only use the fact that a gene was spatially regulated, rather than its actual expression level, to compare gene expression profiles of articular and growth plate cartilage zones. Nevertheless, in spite of these limitations, our bioinformatic and statistical analyses, including principal components analysis, hierarchical cluster analysis, heat map visualization, overlap of spatially upregulated genes between articular and growth plate cartilage zones, and localization of known growth plate cartilage zonal markers in articular cartilage, consistently indicated that, in contrast to our hypothesis, IDZ has an expression profile more similar to that of RZ, whereas the SZ gene expression profile appears to be more similar to those of PZ and HZ. Furthermore, we repeated the manual microdissection to isolate SZ and IDZ of articular cartilage as well as RZ, PZ, and HZ of growth plate cartilage from 10-day-old rat proximal tibias to confirm gene expression patterns by real-time PCR. The expression patterns detected in this new dataset for 12 selected genes were similar to those observed by microarray analysis and bioinformatics using two different microarray platforms, growth plate cartilage zones from 7-day-old rats, and articular cartilage zones from 10-day-old rats, and thus validate the accuracy of our approach. Spatially upregulated genes that overlap between SZ and HZ (Figure 4) included an inhibitor of angiogenesis *Adamts1* [29], as well as matrix metalloproteinases *Mmp9* and *Mmp13*, which are essential for endochondral bone formation [30] but also implicated in the pathophysiology of osteoarthritis [31], [32]. These findings suggest that progenitor cells of articular cartilage may also reside in the deeper layers and that chondrocytes in articular cartilage differentiate toward SZ following a differentiation program that, despite the lack of hypertrophy, has distinct transcriptional similarities with the growth plate chondrocyte differentiation program. Furthermore, these findings are largely consistent with cell lineage tracing studies in mice showing that all the zones of articular and growth plate cartilage originate from collagen type 2-expressing chondrocytes in the cartilaginous condensation [33]–[35].

In order to understand the early transcriptional differences responsible for the divergence of articular and growth plate cartilage we also identified genes that are differentially expressed between IDZ and RZ. Functional pathway analysis implicated biologically relevant pathways including sonic hedgehog (SHH) and bone morphogenetic protein (BMP) activity in RZ. The hedgehog family of proteins, including SHH, is important for normal skeletogenesis, such as articular and growth plate cartilage development [36]. Overexpression of SHH in chondrocytes disrupts cell differentiation, growth plate cartilage organization, and joint cavity delimitation leading to fusion of articular surfaces [37]. BMPs are known to play important roles in endochondral ossification by promoting growth plate chondrocyte proliferation and hypertrophic differentiation [38], [39]. In growth plate cartilage, BMP antagonists *Gremlin*, *Chordin* and *Bmp3* are highly expressed in RZ and *Gdf10* in PZ, whereas BMP agonists *Bmp2* and *Bmp6* are highly expressed in HZ and *Bmp7* in PZ, suggesting a functional BMP gradient, where BMP signaling is lower in RZ and higher in HZ [11]. The analysis also implicated biologically relevant pathways in IDZ, including Role of Osteoblasts, Osteoclasts and Chondrocytes in Rheumatoid Arthritis. Upregulated genes in this pathway include Wnt inhibitory factor 1 (*Wif1*), which is a Wnt receptor inhibitor. This finding makes biological sense because Wnt signaling promotes hypertrophic differentiation and matrix mineralization, events that are absent in healthy articular cartilage [40]. Wnt signaling itself was among the pathways implicated in the difference between gene expressions of IDZ and RZ, where it was relatively more active in RZ (Table S4 in File S1).

In summary, we used manual microdissection, microarray analysis, bioinformatics, and real-time PCR to characterize gene expression patterns in articular and growth plate cartilage and found, contrary to our hypothesis, that the gene expression changes taking place between the IDZ to SZ of articular cartilage have many similarities with those that occur during the differentiation of resting to proliferative and then to hypertrophic chondrocytes in growth plate cartilage. These findings suggest that the SZ chondrocytes of articular cartilage differentiate according to a program that is not completely different from, but instead has distinct similarities to, the hypertrophic differentiation program of growth plate chondrocytes. We also identified genes that are differentially expressed in IDZ of articular cartilage and RZ of growth plate cartilage at the time when these two structures are initially being separated by the secondary ossification center, and these genes implicated hedgehog and BMP signaling, among others, as potential key pathways in the divergence of articular and growth plate cartilage.

## Supporting Information

File S1 **Containing Tables S1, S2, S3, and S4.** Table S1. Differentially expressed genes between superficial and intermediate/deep zones of articular cartilage. Table S2. Signaling pathways implicated by spatially upregulated genes that overlap between superficial and hypertrophic zones, superficial and proliferative zones, as well as intermediate/deep and resting zones. Table S3. Differentially expressed genes between intermediate/deep zone of articular cartilage and resting zone of growth plate cartilage. Table S4. Signaling pathways implicated by differentially expressed genes between intermediate/deep zone of articular cartilage and resting zone of growth plate cartilage.
(XLSX)
